# Probabilistic modelling of developmental neurotoxicity based on a simplified adverse outcome pathway network

**DOI:** 10.1016/j.comtox.2021.100206

**Published:** 2022-02

**Authors:** Nicoleta Spînu, Mark T.D. Cronin, Junpeng Lao, Anna Bal-Price, Ivana Campia, Steven J. Enoch, Judith C. Madden, Liadys Mora Lagares, Marjana Novič, David Pamies, Stefan Scholz, Daniel L. Villeneuve, Andrew P. Worth

**Affiliations:** a School of Pharmacy and Biomolecular Sciences, Liverpool John Moores University, Byrom Street, Liverpool L3 3AF, UK; b Department of Psychology, University of Fribourg, Fribourg CH-1700, Switzerland; c European Commission, Joint Research Centre (JRC), Ispra, Italy; d Jožef Stefan International Postgraduate School, 1000 Ljubljana, Slovenia; e Theory Department, Laboratory for Cheminformatics, National Institute of Chemistry, 1000 Ljubljana, Slovenia; f Department of Biomedical Science, University of Lausanne, Lausanne, Vaud, Switzerland; g Swiss Centre for Applied Human Toxicology (SCAHT), Switzerland; h Helmholtz-Centre for Environmental Research − UFZ, Department of Bioanalytical Ecotoxicology, Permoserstrasse 15, 04318 Leipzig, Germany; i US Environmental Protection Agency, Great Lakes Toxicology and Ecology Division, Duluth, MN 55804, MN, USA

**Keywords:** Developmental Neurotoxicity, Bayesian hierarchical model, Adverse Outcome Pathway, Common Key Event, New Approach Methodology, ADMET, Absorption, distribution, metabolism, excretion, and toxicity, AO, Adverse outcome, AOP, Adverse outcome pathway, BBB, Blood-brain-barrier, BDNF, Brain-derived neurotrophic factor, CAS RN, Chemical Abstracts Service Registry Number, CI, Credible interval CKE, Common key event, CNS, Central nervous system, CRA, Chemical risk assessment, DAG, Directed acyclic graph, DNT, Developmental neurotoxicity, DTXSID, The US EPA Comptox Chemical Dashboard substance identifier, EC, Effective concentration, HDI, Highest density interval, IATA, Integrated Approaches to Testing and Assessment, KE, Key event, KER, Key event relationship, LDH, Lactate dehydrogenase, MCMC, Markov chain Monte Carlo, MIE, Molecular initiating event, NAM, New approach methodology, OECD, Organisation for Economic Cooperation and Development, PBK, Physiologically-based kinetic, P-gp, P-glycoprotein, qAOP, Quantitative adverse outcome pathway, QSAR, Quantitative structure-activity relationship, SMILES, Simplified molecular input line entry system

## Abstract

In a century where toxicology and chemical risk assessment are embracing alternative methods to animal testing, there is an opportunity to understand the causal factors of neurodevelopmental disorders such as learning and memory disabilities in children, as a foundation to predict adverse effects. New testing paradigms, along with the advances in probabilistic modelling, can help with the formulation of mechanistically-driven hypotheses on how exposure to environmental chemicals could potentially lead to developmental neurotoxicity (DNT). This investigation aimed to develop a Bayesian hierarchical model of a simplified AOP network for DNT. The model predicted the probability that a compound induces each of three selected common key events (CKEs) of the simplified AOP network and the adverse outcome (AO) of DNT, taking into account correlations and causal relations informed by the key event relationships (KERs). A dataset of 88 compounds representing pharmaceuticals, industrial chemicals and pesticides was compiled including physicochemical properties as well as *in silico* and *in vitro* information. The Bayesian model was able to predict DNT potential with an accuracy of 76%, classifying the compounds into low, medium or high probability classes. The modelling workflow achieved three further goals: it dealt with missing values; accommodated unbalanced and correlated data; and followed the structure of a directed acyclic graph (DAG) to simulate the simplified AOP network. Overall, the model demonstrated the utility of Bayesian hierarchical modelling for the development of quantitative AOP (qAOP) models and for informing the use of new approach methodologies (NAMs) in chemical risk assessment.

## Highlights

• A developmental neurotoxicity Adverse Outcome Pathway network was simplified.
• Common key events were chosen based on topology analysis and expert judgement.
• Quantification of causal relationships was informed by key event relationships.
• Various types of information were integrated for probability prediction.
• Bayesian hierarchical modelling was applied for hazard identification.

## Graphical abstract

The conceptual framework used to develop the Bayesian hierarchical model. The model comprised three common key events shown in green selected based on topology analysis and expert knowledge to describe developmental neurotoxicity. These served as a basis to define the structure of the quantitative model. Appropriate data were collected to fit the model with the aim of predicting the probability of whether a compound triggers the biological path.

**Figure:**
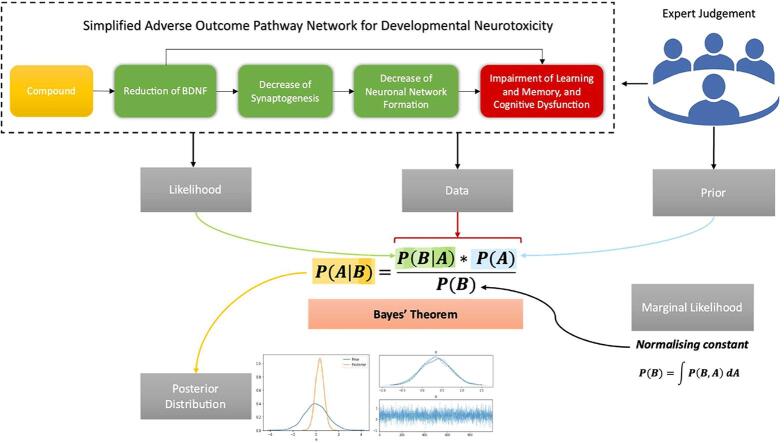

## 1 Introduction

Neurodevelopmental disorders such as impairment of learning and memory, and cognitive dysfunction, are of serious concern due to the health risks and consequences on the developing brain resulting from exposure to exogenous chemicals [1], [2], [3]. The assessment of developmental neurotoxicity (DNT) is not a mandatory requirement in the European Union or the United States of America and is not routinely conducted. However, it may be undertaken when data from developmental and/or reproductive toxicity studies on adult animals indicate a possible concern for neurotoxicity [4]. When testing is carried out, it is based on available DNT testing guidelines using *in vivo* methods [5], [6], [7]. These animal tests are a starting point in deciphering complex endpoints such as DNT. However, with the limitations of *in vivo* testing for DNT [8], there is an opportunity to consider new approach methodologies (NAMs), such as a battery of *in vitro* test methods, omics technologies and *in silico* models as a viable alternative. Whilst NAMs for DNT are not yet standardised or required by regulatory authorities, they can provide valuable mechanistic insights regarding potential developmental neurotoxicants [4], [9], [10], [11].

Frameworks are required to organise information from NAMs to predict complex toxicological endpoints, including neurotoxicity and DNT. A promising approach to organise DNT information and subsequently use the information to develop a predictive model is provided by the adverse outcome pathway (AOP) concept [12]. An AOP represents a formal description of a series of events from the molecular initiating event(s) (MIE(s)), key events (KEs) at corresponding molecular, cellular, and tissue levels to adverse outcomes (AOs) at organ, organism and population levels [12]. Recent progress in the development of qualitative and quantitative AOPs underlines their utility to design appropriate experiments and computational simulations [13], [14]. However, as DNT is a complex process with multiple molecular and cellular paths, no single AOP is able to fully explain this complex event. Thus, a network of AOPs allows for a better depiction of the overall mechanistic understanding of DNT than a single AOP. To illustrate this point, given the limited resources available to quantify biological paths of DNT, identification of common key events (CKEs) that intersect the individual paths can assist in the design of testing strategies and in the computational modelling of data-rich biological events [15]. Such CKEs are characterised by high connectivity located, are centrally within the network of AOPs and are essential to link multiple linear AOPs, i.e., MIE(s) to the AO(s) [16], [17].

Quantitative AOPs (qAOPs) and qAOP networks are increasingly being modelled using probabilistic methods [18], [19]. Bayesian modelling is an approach that fits, and makes inferences from, data using Bayes’ theorem for variables. Bayes’ theorem is a mathematical formula that transforms the prior, i.e., our knowledge about the data before seeing the data, into posterior distributions based on the evidence provided [20], [21]. An advantage of this approach, besides the ease of computing predictions and associated uncertainties relating to variables of interest, lies in the reproducibility of the predictions; once the prior beliefs are defined, similar values will be obtained each time the model is run unless new evidence is added [20], [22].

Given the limited availability and heterogeneity of DNT information, i.e., *in vivo* and *in vitro* data, and the potential for Bayesian machine learning to investigate chemical-induced DNT, the aim of this investigation was to quantify a simplified, reduced version of the AOP network for neurotoxicity developed by Spînu et al. [23]. The main objective was to predict the probability that a compound induces individual CKEs, in addition to predicting the probability of inducing the AO. The analysis took into account potential correlations and causal relations informed by the key event relationships (KERs) and additional information, such as physicochemical, *in silico* and *in vitro* data. This investigation also aimed to explore whether Bayesian hierarchical modelling is fit-for-purpose in chemical risk assessments informed by qAOP models.

## 2 Materials and methods

### 2.1 Simplification of the AOP network

The AOP network for neurotoxicity developed and analysed by Spînu et al. [23] served as a foundation to establish the graphical structure of the quantitative model. Specifically, three CKEs were selected to describe a biological path for DNT representative of the AOP network. These CKEs were amongst those identified by the topology analysis reported by Spînu et al. [23]. The choice of CKEs was, however, also made based on expert judgement to avoid non-specific CKEs such as cell injury/death as well as on data availability that would allow quantification. The expert decisions were made during a workshop entitled “*e-Resources to Revolutionise Toxicology: Linking Data to Decisions*”, held at the Lorentz Center (Leiden, The Netherlands) in October 2019 [24]. The biological path chosen describes the reduction of brain-derived neurotrophic factor (BDNF) that leads to a decrease of synaptogenesis and a decrease of neural network formation and function involved in DNT (Fig. S1). Additionally, this biological path is supported by the description of the AOP ID 13 (https://aopwiki.org/aops/13; [25]) and assessed for its availability in the Organisation for Economic Cooperation and Development (OECD) AOP-Wiki Knowledge Base in Table S1. Importantly, the AOP network reported by Spînu et al. [23] was not a directed acyclic graph (DAG), i.e., a graph with no cyclic paths (no loops). However, the simplified version used in this investigation represented a DAG to allow for the formulation of a causal hypothesis and methodological approach. Such a combination of expert judgement and topology analysis can provide a foundation to establish quantitative models.

### 2.2 Data description

Two *in vitro* studies [26], [27] that tested compounds for their DNT potential were chosen as primary data sources. The compounds tested from the two studies were merged into a single list (e.g., nine pairs of compounds, tested as different forms of salts, were combined) and aligned with information referring to the compound name, Chemical Abstracts Service Registry Number (CAS RN), simplified molecular input line entry system (SMILES) string and the US EPA Comptox Chemical Dashboard substance identifier (DTXSID). In total, 88 compounds served as a starting point to collect additional information to improve the modelling. The list contained different types of compounds: pharmaceuticals, pesticides and industrial chemicals. The two *in vitro* studies differed in the concentration ranges used, type of viability assay applied, plating densities of the cells, calculation of the effective concentration (EC), and the exposure period (five days vs 12 days). Synaptogenesis was measured by a battery of high content imaging and microplate reader-based assays, while the viability was based on the number of cells per field or determined in sister plates using a luminescent assay [27]. Neural network activity was measured on day 12 by the microelectrode array recordings and the viability by two assays, the total lactate dehydrogenase (LDH) release and Alamar blue assay [26]. Also, valproate was tested in two concentration ranges in both studies: lower and higher. The range of higher concentrations showed response/activity in both studies and, thus, the EC_x_ values of the range of higher concentrations tested, and associated responses, for valproate were retained. The difference in the concentration range and time of effect shows that impacts on synaptogenesis are observed at lower levels than impacts on neural network formation. This concentration–response concordance fits the expected pattern for a causal relationship between the two key events in an AOP [28].

The compounds were assigned as positive or negative for DNT induction based on the *in vivo* studies summarised and evaluated in a literature review by Mundy et al. [29]. In the latter paper, the authors applied rigorous selection criteria to 408 chemicals, concluding that 97 showed evidence of developmental neurotoxicity. Of these 97 DNT-positive chemicals, 21 had evidence in human studies. The compounds were assigned positive/negative for the reduction of BDNF based on a literature review performed to evaluate the peer-reviewed publications that studied the impact of compounds on this effect (Supplementary material, Table S2). Given that at present there is no standardised protocol for assessing the inhibition of BDNF available and thus, no existing datasets, the main selection criteria of the peer-reviewed publications included any available *in vitro* and/or *in vivo* studies that showed a decreased or increased level of BDNF for each chemical included in the test set. Compounds were further classified as active/inactive for the CKEs relating to the decrease of synaptogenesis and neural network formation; this was based on the selectivity and specificity identified from the corresponding *in vitro* studies taking into account cell viability and the results of toxicity assays.

Other data collected included the calculated logarithm of the distribution coefficient (LogD, pH = 7.4), a measure of lipophilicity for ionizable compounds; blood–brain-barrier (BBB) permeability; and the capability to bind to the P-glycoprotein (P-gp) transporter, i.e., if the compound acts as a P-gp inhibitor or substrate, or is (non-)active against P-gp. Predictions for BBB permeability and P-gp interactions were made using *in silico* models based on curated SMILES [30], [31], [32]. BBB permeability is essential for understanding whether a compound crosses into, and has the possibility to act on, the central nervous system (CNS) [33]. P-gp is a transmembrane protein belonging to the ATP-binding cassette family of transporters (ABC-transporters), highly associated with the absorption, distribution, metabolism, excretion, and toxicity (ADMET) properties of compounds. P-gp may contribute to a decrease in toxicity by eliminating the compound from cells and preventing its intracellular accumulation [30].

**Fig. 1:**
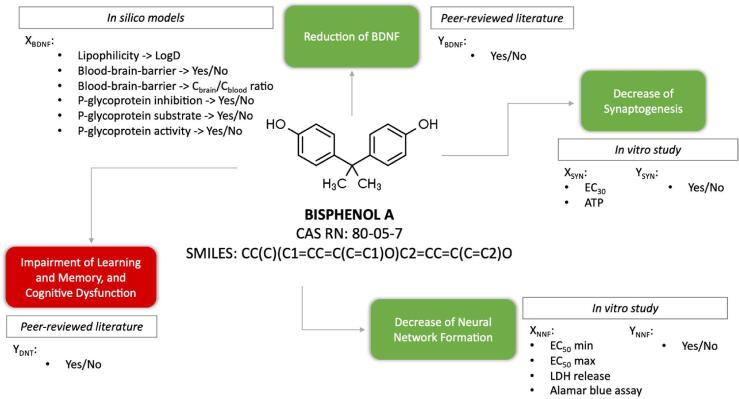
Types of information collected for model development exemplified for bisphenol A. This figure illustrates how different streams of data can be integrated for causal predictions to complement the information on key events. The full data set is provided in Table S3 in the supplementary material. The EC_x_ values were extracted from the corresponding *in vitro* studies as published.

The final data set contained missing information given by the *in silico* models for e.g., inorganic compounds, and there were compounds with no available evidence in the literature for the reduction of BDNF. In addition, inactive compounds in any of the *in vitro* assays were treated as missing information. Eight compounds contained complete details for this biological path. The final data set is provided in Table S3 in the supplementary material. The types of data utilised for modelling DNT are illustrated in Fig. 1, while further details are provided in Table 1.

**Table 1:** Types of data and their sources collected for the development of the Bayesian hierarchical model. The table describes all variables, i.e., predictors and outcomes defined as features included in the model for the type of data and performance where applicable. See also Tables S1-S2 in the supplementary material.

**Feature**	**Description/Relevance**	**Data Type**	**Performance**	**Source**
Chemical Name	The names used to define the compounds tested in both *in vitro* studies.	Not Applicable	Not Applicable	[26], [27]
CAS RN	Chemical Abstracts Service Registry Number associated with the tested compounds used to identify and track them during the modelling.	Not Applicable	Not Applicable	[26], [27]
DNT Classification	Each compound was classified as either positive, known or potential inducing DNT/negative, safe or without evidence for inducing DNT based on *in vivo* studies.	Binary, i.e., positive (i.e., associated with DNT) or negative	Not Applicable	[26], [27], [29]
LogD	The logarithm distribution coefficient calculated based on the compounds’ SMILES strings.	Continuous, unitless values	Not Applicable	ChemSpider database [34]
BBB	Each compound was classified for its capability to permeate the blood–brain-barrier (BBB) based on curated SMILES. Predicting BBB permeability means indicating whether compounds pass across the BBB. Compounds that cross the BBB have the potential to be CNS-active, whereas compounds that do not cross are expected to be CNS-inactive.	Binary, i.e., positive (BBB permeable) and negative	The *in silico* model available in admetSAR v2.0 has the area under the curve (AUC) with a range from 0.625 to 0.99.	Literature review Online BBB Predictor v.0.9 admetSAR v.2.0 [32], [35]
Cbrain/Cblood	*In vivo* blood–brain-barrier penetration represented as BB = [Brain]/[Blood], where [Brain] and [Blood] are the steady-state concentration of radiolabelled compounds in the brain and peripheral blood. High absorption to CNS had a value of more than 2.0, medium absorption: 2.0–0.1, and low absorption: less than 0.1. The predictions are based on *in vivo* data on rats.	Continuous, unitless values	The QSAR model of Ma et al. [31] had R = 0.955 with s = 0.232, used by PreADMET v.2.0 to model the predictions.	PreADMET v.2.0 [31], [36]
P-glycoprotein Status	Each compound was classified based on curated SMILES as a substrate or not, inhibitor or not, active or inactive for P-glycoprotein (P-gp) transporter using an *in silico* model.	Binary, i.e., yes or no for a compound acting as a substrate or an inhibitor, or activity against P-gp	The non-error rate and the average precision was 0.70 for the external validation set.	[30]

**Feature**	**Description/Relevance**	**Data Type**	**Performance**	**Source**
Reduction of BDNF	Each compound was classified as either positive, inducing the reduction of BDNF levels, or negative based on a literature search for *in vivo* studies, e.g., in rats and mice, and/or *in vitro* studies, e.g., human neuroblastoma SH-SY5Y cell line, embryonic mouse hypothalamus cell line.	Binary, i.e., positive (evidence showing alterations of BDNF), or negative	Not Applicable	Literature review of historical and peer-reviewed studies that evaluated compounds for their BDNF reduction potential
Decrease of Synaptogenesis	Selectivity and potency of a chemical were kept as classified in the reference based on results for viability and effective concentrations in rat primary cortical cells.	Categorical, i.e., inducing or not alterations of synaptogenesis	The battery assay had a sensitivity of 87% and a specificity of 71%.	[27]
Synaptogenesis Viability	The amount of ATP present in each well was calculated to assess compounds for their viability in rat primary cortical cells.	Continuous	Not Applicable	[27]
Synaptogenesis Activity, EC_30_ (μM)	30% change compared to control expressed as an effective concentration EC_30_ (μM) for puncta per total dendrite length (the most sensitive endpoint) measured in rat primary cortical cells for five days using an imaging assay.	Continuous	Not Applicable	[27]
Decrease of Neural Network Formation	Selectivity and potency of a chemical were kept the way the reference classified a compound based on results for viability and effective concentrations in rat primary cortical cells.	Categorical, i.e., inducing or not alterations of neural network formation	The model had a mean accuracy of 80.2%.	[26]
Neural Network Formation Viability	Total lactate dehydrogenase (LDH) release upon cell lysis and Alamar blue assay were used to assess compounds for their viability in rat primary cortical cells.	Continuous	Not Applicable	[26]
Neural Network Formation Activity, EC_50_min and EC_50_max (μM)	50% change compared to control expressed as an effective concentration EC_50_ (μM) with minimum and maximum values of all 17 parameters measured in rat primary cortical cells over 12 days using microelectrode array (MEA) recordings.	Continuous	Not Applicable	[26]

### 2.3 Exploratory data analysis

Exploratory data analysis was applied to analyse the data collected and to summarise their main characteristics, including the types of variables (e.g., continuous or discrete); the shape of the empirical distributions (i.e., histogram) of dependent variables; correlations between the variables using the Pearson correlation coefficient; the distribution of the missing values; chemical characteristics described by physicochemical properties associated with the categorical classification, i.e., positive/negative, of the CKEs and DNT; and the presence of (un)balanced categories in the dataset. The analysis was conducted to help choose the appropriate priors and define regression models and the overall strategy for computational modelling.

### 2.4 Bayesian hierarchical approach

A Bayesian hierarchical model is a combination of sub-models in which the parameters are informed by a common hyperprior. The model is structured into exchangeable levels/groups, e.g., CKEs, categories of chemicals, taking into account the inter-independencies and interactions between those groups, leading to improved inferences [20], [37]. This type of model is ideal for the complex structure of data involving multilevel organisation, similar to that implied for the simplified AOP network for DNT.

**Fig. 2:**
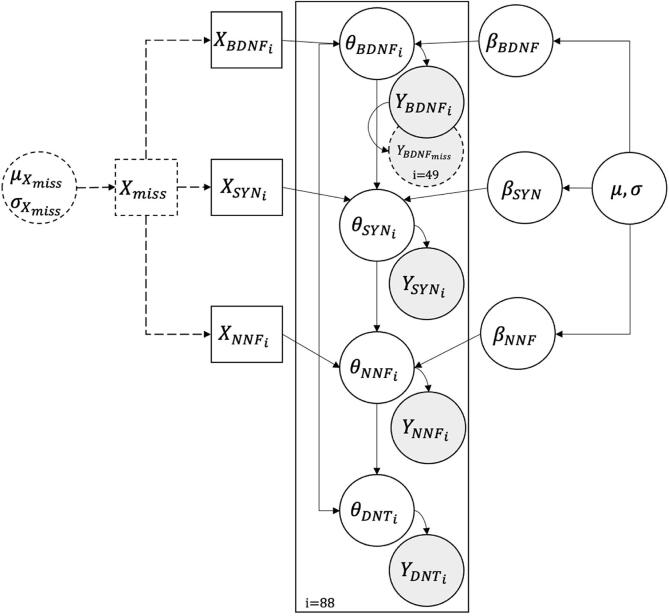
A simplified graphical representation of the Bayesian hierarchical model utilised to assess individual compounds for their DNT induction potential. The model follows a specific biological path in the AOP network for DNT. The dotted lines represent the imputation step of the missing values, which was conducted either from the prior distribution for X or from the posterior distribution for Y. BDNF: reduction of brain-derived neurotrophic factor; SYN: decrease of synaptogenesis; NNF: decrease of neural network formation; DNT: developmental neurotoxicity; miss: missing values; i: number of compounds; X: predictors, independent variables; Y: outcomes, dependent variables; β,μ,σ,θ: parameters of the model.

A single nested partial pooled Bayesian hierarchical model was formulated as outlined in Fig. 2. It consisted of nine unknown parameters: the two hyperpriors μ and σ, three priors βs, and data likelihoods that parameterised the four θs. In a Bayesian framework, all the unknown parameters have predefined distributions representing our belief before evaluating the data, and these are estimated from the data [20]. Because of the hierarchical type of modelling, the parameters were sampled from a common global distribution given by the hyperpriors that were defined as weakly-informative on the entire covariance matrix. Weakly-informative hyperpriors (i.e., less restrictive and/or diffuse) were chosen because of the data sample size additionally minimising the impact on the posterior [21]. The tilde sign “∼” indicates the type of distribution the parameter was generated from. Herein, a common mean μ and standard deviation σ were defined to describe the global distribution of the entire dataset and were generated from normal and half-normal distributions. This represents the single nested part of the model.

$$\mu \sim \;Normal\left(0,\;0.1\right)$$

$$\sigma \sim \;HalfNormal\left(1\right)$$

Each β varied per CKE and, thus, the group-level CKEs were generated from a normal distribution and shrunk towards the hyperpriors specified above. This describes the partial-pooled part of the model.

$$\beta_{\mathrm{BDNF}},\beta_{\mathrm{SYN}},\beta_{\mathrm{NNF}}\sim \;Normal\left(\mu ,\sigma \right)$$

The missingness was treated by Bayesian imputation. Each missing data point in the design matrix X was masked in advance to indicate missing information that would subsequently be estimated probabilistically. If X predictors contained missing data, the imputation was sampled from the prior distribution as shown below, while missing Y outcomes were imputed from the posterior predictive distributions. Weakly-informative priors were defined to sample the missingness for all CKEs.

$$\mu_{\mathrm{Xmiss}}\sim \;Normal\left(0,\;0.1\right)$$

$$\sigma_{\mathrm{Xmiss}}\sim \;HalfNormal\left(1\right)$$

$${P(X}_{\mathrm{miss}}\left|X_{\mathrm{CKE}}\right)\sim \;Normal\left(\mu_{\mathrm{Xmiss}},\;\sigma_{\mathrm{Xmiss}}\right)$$

The matrices of X predictors and the β parameters were then multiplied to obtain the linear prediction θs which represent the likelihood of observing the CKEs (0 or 1). The β parameters describing each CKE were estimated from data. Besides the multiplication, the causal relationship is described as a set of linear regressions with the latent variable, e.g., Y of CKE 1 reduction of BDNF, as a predictor that progresses into the next linear regression, e.g., Y of CKE 2 decrease of synaptogenesis to follow the DAG structure of the simplified AOP network. Importantly, the likelihood for the AO of DNT was informed solely by the sum of the likelihood of the CKEs. The subscript i index indicates that the probability was estimated for each compound i.

$$\theta_{{\mathrm{BDNF}}_{i}}=\beta_{\mathrm{BDNF}}*X_{\mathrm{BDNF}}$$

$$\theta_{{\mathrm{SYN}}_{i}}=\beta_{\mathrm{SYN}}*X_{\mathrm{SYN}}+\theta_{{\mathrm{BDNF}}_{i}}$$

$$\theta_{{\mathrm{NNF}}_{i}}=\beta_{\mathrm{NNF}}*X_{\mathrm{NNF}}+\theta_{{\mathrm{SYN}}_{i}}$$

$$\theta_{{\mathrm{DNT}}_{i}}=\theta_{{\mathrm{BDNF}}_{i}}+\theta_{{\mathrm{SYN}}_{i}}+\theta_{{\mathrm{NNF}}_{i}}$$

The three CKEs were generated from a Bernoulli distribution of deterministic relationships estimated from the data. The AO was also generated from a Bernoulli distribution of the deterministic relationship that summed up the logistic regression of all θ s that described the CKEs independently.

$$Y_{{\mathrm{BDNF}}_{i}}\;\sim Bernoulli\left(\theta_{{\mathrm{BDNF}}_{i}}\right)$$

$$Y_{{\mathrm{SYN}}_{i}}\;\sim Bernoulli\left(\theta_{{\mathrm{SYN}}_{i}}\right)$$

$$Y_{{\mathrm{NNF}}_{i}}\;\sim Bernoulli\left(\theta_{{\mathrm{NNF}}_{i}}\right)$$

$$Y_{{\mathrm{DNT}}_{i}}\;\sim Bernoulli\left(\theta_{{\mathrm{DNT}}_{i}}\right)$$

Model building and inference was carried out using PyMC3 version 3.9.3 [38]. The posterior distribution was sampled using Markov chain Monte Carlo (MCMC) methods (No-U-Turn Sampler (NUTS), a dynamic variant of Hamiltonian Monte Carlo). Imputation for missing values was performed automatically during inference. The Bayesian credible interval (CI) of 95%, also known as the highest density interval (HDI), an interval within which an unobserved parameter value falls with a particular probability, was applied. A 95% credible interval has the upper and lower 2.5th percentiles of the posterior distribution as its bounds.

### 2.5 Model fitting

The Bayesian hierarchical model had 783 parameters in total for a dataset of 88 compounds that described the CKEs as well as each chemical independently (Supplementary material, Table S10). The number of parameters posed a risk of overfitting; this means that a model learns too much from the sample [20], [22]. In a Bayesian framework, a significant source of overfitting is the choice of priors that have a vital role in normalising the likelihood functions and thus being propagated throughout the model [20], [22]. Hence, the risk of overfitting depends on both the structure of the model and the size of the sample. To reduce the risk of overfitting, we opted to shrink the β parameters towards a common hyperprior (μ and σ) that controlled their distribution and, hence, opted for a partial pooling of the β parameters to produce estimates for each CKE and the AO, and we chose weakly-informative hyperpriors and priors to regularise the inferences. By doing so, a reasonable compromise is obtained between the bias and the variance in the estimated parameters.

To show that the model is data-driven and not significantly influenced by the choice of hyperpriors, a sensitivity analysis was performed. Two additional weakly-informative hyperpriors were chosen. The comparison of the models was conducted for three different metrics including:

(1) The Pareto-smoothed importance sampling leave-one-out cross-validation (PSIS-LOO-CV) statistic, which measures the predictive accuracy of a Bayesian model by fitting a Pareto distribution to the upper tail of the distribution of the importance weights to estimate pointwise predictive density [39].
(2) The Widely applicable information criterion (WAIC), which is an alternative approach for estimating the out-of-sample expectation based on pointwise calculations considered to be asymptotically equal to leave-one-out cross-validation [39].
(3) The Brier score [40], which measures the mean squared difference between the predicted probability and the actual outcome and lies between zero and one. The smaller the Brier score, the smaller the difference between the actual and predicted values is and the more accurate the prediction is.

$$\mathrm{Brier}\;score\;\left(BS\right)=\frac{\sum_{i=1}^{N}{\left({\mathrm{predicted}}_{i}-{\mathrm{actual}}_{i}\right)}^{2}}{N}$$

In addition, model fitting was evaluated for its convergence to the target distribution using several statistical measures. The Gelman-Rubin diagnostic, also known as the R-hat statistic, measures how similar different MCMC chains are, i.e., within and between chains and thus whether the chains converge to the same distribution [41]. The Monte Carlo standard error is another measure of the accuracy of the chains, given by the posterior standard deviation divided by the square root of the number of the effective samples [20]. The smaller it is, the closer the posterior mean is expected to be to the actual value. The effective sample size estimates the independent sample because samples are typically autocorrelated within a chain and can increase the uncertainty in estimates [20]. The effective sample size should be at least equal to the actual number of samples.

### 2.6 Model performance

Four different metrics were employed for the evaluation of the model performance:

(1) Sensitivity, also referred to as true positive rate, measures the ability of a model to correctly detect a positive sample as positive:

$$\mathrm{sensitivity}=\frac{\mathrm{TP}}{\mathrm{TP}+FN}$$

(2) Specificity, also referred to as true negative rate, measures the ability of a model to find all negative samples:

$$\mathrm{specificity}=\frac{\mathrm{TN}}{\mathrm{TN}+FP}$$

(3) Accuracy measures the number of correctly predicted data points out of all the data points:

$$\mathrm{accuracy}=\frac{\mathrm{TP}+TN}{\mathrm{TP}+FP+TN+FN}$$

where FN stands for false negatives, FP stands for false positives, TN stands for true negatives, TP stands for true positives.

(4) Balanced accuracy quantifies the average of sensitivity and specificity and is robust against data imbalances between the two classes.

These metrics were reported for two CKEs and the AO. It was not possible to evaluate the CKE reduction of BDNF because of the presence of the missing information about the activity of the studied compounds for this protein.

## 3 Results

The exploratory data analysis allowed for a better understanding of the data collected and utilised for the Bayesian analysis (Supplementary material, Figs. S2–S5). The statistical parameters of the model did not show the presence of any divergent transitions to approximate the posterior distributions (Supplementary materials, Figs. S6-S7 and Table S10). The estimates of the hyperpriors and priors are summarised in the supplementary material, Fig. S8.

**Fig. 3:**
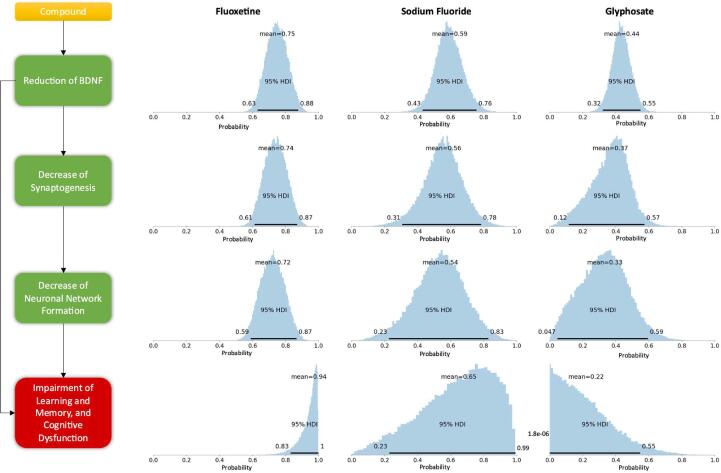
Visualisations of the predictions for three compounds: fluoxetine, sodium fluoride and glyphosate to show the relationships between the compounds and CKEs and the AO following the structure of the simplified biological path for DNT. The shaded blue distribution represents the predicted severity of the CKEs and the AO as well as the uncertainty in the prediction for the 95% highest density interval (HDI). For a complete overview of the results of all compounds, the reader is referred to Fig. S9 in the supplementary material. (For interpretation of the references to colour in this figure legend, the reader is referred to the web version of this article.)

The posterior distributions, (i.e., likelihood, posterior densities) and posterior predictive distributions were obtained from the Bayesian analysis. The posterior distributions represent the evidence provided by the data combined with the prior that incorporates our knowledge before analysing the data. The posterior probability distributions for each CKE, including the AO, are summarised in Fig. S9. The Bayesian CI was broader for compounds with missing information. Thus, the Bayesian CI quantifies the uncertainty given by the sources of variability and missingness. For instance, fluoxetine with information across all levels had a 95% HDI of 0.83–1.0 with a mean probability of 0.94 for the induction of DNT (Fig. 3). In contrast, sodium fluoride with missing data for the CKEs had a 95% HDI of 0.23–0.99 with a mean probability of 0.65 for the induction of DNT (Fig. 3). Glyphosate, a known negative DNT, had a very low 95% HDI with a maximum of 0.55 with a mean of 0.22 for the induction of DNT (Fig. 3).

**Fig. 4:**
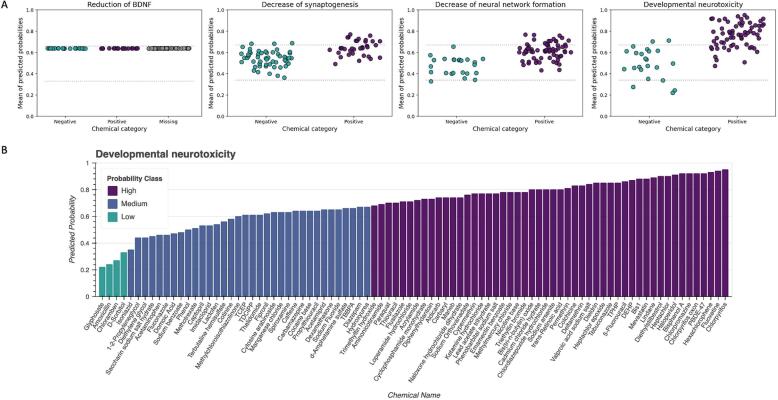
**The results of the posterior predictive probabilities.** A. The distribution of the posterior probabilities of the three CKEs and the AO for positive and negative compounds, and compounds with a level of missingness for the CKE reduction of BDNF. It shows how the mean of the predicted probability of compounds was clustered and distributed based on the two thresholds to describe a low, medium and high probability for the induction of the corresponding CKE and AO. A detailed graphical representation is shown in Supplementary material, Figs. S10–S12. B. Predicted probabilities of compounds for the induction of developmental neurotoxicity. The predicted probabilities are colour-coded based on two thresholds estimated from the results set to group the compounds for their low, medium and high probability. Compounds were listed in the order of increasing probability.

The posterior predictive distributions are given by the binary classification of compounds that were analysed against the likelihood. The binary classification was informed by the literature review for the CKE reduction of BDNF and by the *in vitro* studies for the other two CKEs and expressed as a Bernoulli distribution to describe the outcome in a Boolean way, as explained in the Materials and methods section. Two thresholds derived from the results of the predicted posterior distributions were used to classify the compounds for the low, medium or high probability of inducing a CKE and the AO. Such a classification might be used for screening and prioritisation purposes. All compounds with a probability between 0.59 and 0.60 for posterior predictive distributions were classified overall as having a medium probability of inducing a reduction of BDNF (Supplementary material, Fig. S10). This might be a result of the imputation method chosen to deal with the missing information. The results for the decrease of synaptogenesis showed a probability between 0.36 and 0.76 and as such medium and high levels of inducing this CKE (Supplementary material, Fig. S11). For the decrease of neural network formation, the posterior predictive probability was between 0.33 and 0.77 for all compounds that were classified as having low, medium and high levels of probability to induce this CKE (Supplementary material, Fig. S12). An overview of the distribution of the predicted posterior probabilities is shown in Fig. 4.A. The predictions for the AO, which is of most interest for e.g., regulatory decision making, helped to classify the compounds into the three levels (Fig. 4.B). Relatively few compounds had a low level of probability (5% of compounds), and most of the compounds had a high level of probability of inducing DNT (57% of compounds). This unbalanced classification is explained by the initial list of compounds for which 74% of compounds were known to be positive for DNT *in vivo* (in humans or animals). The full results for the prediction of CKEs and the AO are provided in Tables S6–S9 in the supplementary material.

The sensitivity analysis underlined that weakly-informative priors did not have a significant influence on the model, which is instead data-driven (Table S11). The overall performance of the model is summarised in Table S12 with an accuracy of 76% for the prediction of DNT. There were several misclassifications due to a number of reasons including the quality of the data used for modelling and the level of the missing information associated with the CKEs. For example, a posterior predictive probability of 0.71 was obtained for loperamide hydrochloride, which was misclassified with a high level of probability for inducing DNT. The potential reasons for these misclassifications may be in part because of (1) the *in silico* model used for the prediction of P-gp, which classified the compound as an inhibitor, substrate and active, (2) the absence of data for the CKE of reduction of BDNF, (3) the *in vitro* assays that identified it as active, (4) the chosen threshold for classification. However, the large CI associated with the predicted mean value of 0.36–0.99 emphasises the caution required in making decisions for this compound.

## 4 Discussion

The AOP paradigm has provided an opportunity to organise data and information for over a decade. AOPs have developed from the original “linear” concept exemplified by Ankley et al. [12] into networks that better represent the complex ways in which physiology can be perturbed, resulting in adverse outcomes. There are a number of ways to translate the knowledge captured by AOPs and AOP networks into practical tools for risk assessment. This study has developed a qAOP, in this case derived from a simplified AOP network for DNT. Simplification of complex networks, illustrated in this case using graph theory, is vital for identifying the most relevant key events and key event relationships underpinning a complex AO such as DNT.

The approach for modelling the AOP network was based on Bayesian modelling. There are three ways to assign probabilities in a Bayesian model depending on the research question, available evidence and mathematical approach: (1) making subjective assessments (expert judgement), (2) using empirical probabilities based on observed data, and (3) constructing a parametric probability model [20]. The Bayesian hierarchical model developed herein represents a parametric model.

Bayesian machine learning has been increasingly applied in toxicology using the parametric approach, including: improving the modelling of physiologically-based kinetic (PBK) models of inter- or intra-individual variability across a population [42], [43]; assessment of acute kidney injury [44]; evaluation of organ weight toxicity [45]; and a combination of Bayesian statistics and deep learning for the investigation of hepatotoxicity [46]. Bayes nets, also known as Bayesian networks or belief networks, that use conditional probability tables (CPTs) have been proposed as an option for the probabilistic quantification of AOPs [19]. Examples of such modelling include quantification of an AOP network in ecotoxicology [47], evaluation of steatosis under different chemical exposures [48] and the assessment of skin sensitisation potency [49].

A qAOP model is not only knowledge-based but also data-driven. Thus, initiatives that focus on data generation, collection and quality control are crucial for the development of qAOPs. A good example of a data driven approach to support DNT evaluation is the collaborative Developmental Neurotoxicity Data Integration and Visualisation Enabling Resource (DNT-DIVER) project of the National Toxicology Program (NTP) of the National Institute of Environmental Health Sciences (NIEHS) [50]. However, the selection of chemicals for DNT testing might be based on *in vivo* mammalian studies. The requirement for reliable and meaningful data is especially relevant for the mechanistic understanding of the endpoints related to DNT. Thus, the development and use of *in vitro* models could facilitate the generation of relevant and reliable mechanistic data as indicators of DNT, especially when data have been produced under good cell culture practice [51], [52], [53]. For example, a human cell‐based DNT *in vitro* testing strategy has been proposed and evaluated for both the purposes of screening and prioritisation, and hazard characterisation [54]. Such testing strategies and underlying screening libraries can support the development of causal models and build trust in their use, allowing for the incorporation of further levels of detail than those included in our model. For example, the size and composition of the dataset used to develop the model is an important consideration. Even though the compounds considered in this analysis are chemically diverse, there is a need to expand the chemical space to assess new substances.

The model proposed herein analysed a series of compounds for a simplified biological path predicting the probability of inducing each key event. The model represents an attempt to describe a complex endpoint on the basis of limited NAM data. The best predicted event was the AO of DNT (Supplementary information, Table S12). This underlines how the progressive addition of information in the hierarchical model leads to improved predictions, and ultimately, better-informed decisions.

A Bayesian model applied to toxicology should answer causal, rather than purely associative, questions that cannot be computed from data alone. Causal inference can be defined as a way of predicting what would happen, or what might have occurred, to produce an outcome Y given a set of predictors X as a result of a treatment, intervention or exposure Z [55]. Causal effects can take both linear and nonlinear functions. In our model, the causal effects informed by the KERs between the CKEs were treated as linear functions for simplicity. The OECD coordinates an international effort making available systematic knowledge on AOPs. The AOP Knowledge Base plays a pivotal role in supporting research projects and improving the prediction of toxicity in humans [56], [57]. For example, the weight of evidence of KERs can be used to evaluate the reliability and biological relevance of the quantitative predictions. However, in practice, few AOPs exist despite the abundant and increasing mechanistic toxicological knowledge in the scientific literature. Those that do exist often do not provide sufficient quantitative understanding of KERs for many practical applications. Hence, the currently published AOPs represent an incomplete mechanistic understanding of DNT. This means that qAOP models for DNT could equally be based on other MIEs and CKEs – there is no single modelling solution. With this in mind, it is important to underline that the model presented herein is intended to illustrate a strategy for exploiting NAM-generated data relevant to DNT assessment. The proposed modelling strategy is centred on the application of Bayesian machine learning for the development of causal models.

Modelling a qAOP in a Bayesian (hierarchical) manner has several advantages, including:

1. A qAOP model must allow for an objective scientific evaluation of the potential toxicity of chemicals [14]. A Bayesian parametric model aims to determine the posterior distribution for the model parameters, allowing for the quantification of a response, or the effect of one or more KEs in a probabilistic manner. The associated credible interval incorporates the uncertainty of both dependent and independent variables and the different sources of variability. This is especially useful in data-sparse situations such as DNT assessment. Thus, the output of a Bayesian model is more informative than the single best estimate provided by a frequentist model, i.e., “statistically significant” or “non-significant”. As such, it contributes to the paradigm shift in statistical thinking and decision-making called by Amrhein et al. [58] while leading to a transparent, traceable, reproducible and reliable assessments.
2. There is a demand for strategies that better integrate the variety of information sources, including NAMs, available in risk assessment. The aim is to achieve an informed assessment and a structured decision-making process [59], e.g. via Integrated Approaches to Testing and Assessment (IATA) for DNT [8], [60], [61]. In the context of IATA, a Bayesian model can accommodate any type of data useful for assessing chemicals. For example, the combination of *in vitro* information and physicochemical properties can inform better toxicity predictions associated with DNT as presented herein. It can also include missing information, and can cope with the complexity of mechanistic knowledge. Thus, the Bayesian modelling approach offers an understanding of the likelihood of effects and the level of perturbations at different biological levels. It also represents a means to determine whether the available information is sufficient to address a question, and what kind of additional information might be needed. It can also help to screen a large number of compounds and identify tailored toxicological tests for individual compounds or mixtures.
3. A Bayesian hierarchical model is an extension of regression in which data are structured in groups and coefficients can vary by the group [37]. Consequently, hierarchical models can be used for a variety of inferential objectives, including causal inference, prediction and descriptive modelling. For example, the causal inference herein was simulated using linear regressions and the prediction aim was achieved by the two-class logistic regression of the dependent variables implemented. It was also able to capture the variations between the CKEs and the interactions between the variables to predict the effects of individual compounds. The hierarchical approach is better than treating each CKE independently since the data from different CKEs inform one another meaningfully. Under-represented categories of chemicals borrow strength from well-represented chemicals and, thus, the hierarchical approach can deal with the unbalanced classifications under a unified statistical framework. This is especially useful when the datasets are too small to be analysed separately, as is the case with DNT. For example, sodium orthovanadate, which is a known positive *in vivo,* had a predicted probability of 76% to induce DNT because of the hierarchical structure even though it had missing information.

A drawback of Bayesian models is often considered to be the subjectivity of priors. Even though the selection of priors is always debatable, ultimately, the role of priors is to improve the predictions, e.g., the process of data shrinkage towards a prior group-mean to represent the common group distribution [62]. Besides, the AOPs themselves are subjective representations of adverse effects, and hence, there are other subjective elements in the model building process, as argued by van de Schoot et al. [21]. A transparent reporting standard for the selection of data and modelling choices, including assumptions made, would allow for objective evaluation and potential regulatory acceptance of such types of computational models [63]. A list of potential sources of uncertainties that had an impact on the model outcome was assessed (Table 2).

**Table 2:** Qualitative assessment of sources of uncertainties characteristic to the model proposed herein.

**List of potential sources**	Uncertainty degree*	**Reasoning**
***Conceptual model***		
Causal structure	High	The causal links were inferred from well established AOPs, even though there may be other (as yet unknown) causal links. The causal structure does not fulfil all Bradford Hill criteria and should therefore be considered with caution.

***Input data***		
*In vitro* studies	High	The model is data-driven, however, the compiled data set is not ideally suited for modelling purposes (i.e., it was not specifically designed to evaluate computationally such a hypothesis). The variability given by the *in vitro* studies underlines the need for a battery of *in vitro* tests to allow for screening of compounds for their multiple neural activities and key events described within the AOP network.
*In silico* information	High	Limited applicability domain (organics) of the *in silico* models excludes metals and inorganics, which represented a source of missingness.

***Quantitative approach to modelling***		
Probabilistic modelling	Medium	A parametric model was developed. Such a model combined with a subjective assessment type of probabilistic model might lead to a better-informed prediction and increase the trust in its use.
Choice of priors	Low	Weakly-informative priors were chosen with little influence on the posterior probabilities. This is also shown by the sensitivity analysis for exploring three hyperpriors.
Mathematical approach	Medium	Linear and logistic regressions were defined to describe the causal structure. It did not account for temporal dynamics, ADMET, kinetics and types of exposure (acute vs chronic).
Model robustness	Low	Statistical parameters showed the model converged well.
Imputation method	Medium	Prior-based imputation is very informative especially in a hierarchical type of Bayesian model that helps to inform each of the CKEs. Posterior-based imputation led to an almost uniform distribution of posterior predictive probabilities for the reduction of BDNF, a CKE with this type of information missing. Such imputation might suit better multi-classes instead of a binary problem (e.g., proportional odds).
Model performance	High	Several metrics are available with few specifically developed for Bayesian models. The reporting is more important than the selection of such metrics, in addition to making the model accessible. Model performance can have an impact on its future applications.

***Model applicability***		
Uncertainty metrics for outputs	Low	Mean and credible intervals are informative, which is an advantage of the probabilistic approach.
Applicability domain	High	The chemical diversity, in comparison with *in vivo* outcomes, limits the model domain.

* Low – very little impact on the predicted probabilities; Medium – a relatively moderate level of influence on the predicted probabilities; High – a strong influence on the model outcome.

Further modelling efforts should also consider ADMET, kinetics and types of exposure (acute vs chronic). Since DNT effects occur primarily in the offspring following exposure (of the parent) to chemicals, it is essential to study metabolism and other kinetic properties of chemicals, e.g. by using pregnancy-specific PBK models. In other words, once other important kinetic and dynamic types of information, as well as tools to facilitate this understanding, become available, the Bayesian model could be updated accordingly and/or coupled with a PBK model. Importantly, the biological path studied herein does not include any MIEs of the initial AOP network. Linking the Bayesian model to quantitative structure–activity relationship (QSAR) models that evaluate the MIEs could expand its applicability domain.

Today, chemical risk assessment (CRA) tends to be pragmatic, with different methods being combined within an IATA [61], [64]. The aim is to go beyond the existing *in vivo* models towards a better-informed and improved assessment of human health and environmental risks resulting from chemical exposures. Thus, there is momentum to bring CRA up to date with modern technologies and (computational) tools such as qAOPs. Shifting CRA towards probabilistic thinking allows details regarding the level of uncertainty and confidence to be accommodated in the decision-making process. Therefore, a Bayesian hierarchical model can contribute to the paradigm shift towards a mechanistically-driven assessment in modern toxicology and translate a qualitative AOP into a quantitative computational predictive model for potential use in CRA. This study has shown that a qAOP model can serve as a bridge between NAMs and the current regulatory landscape that can help to translate the new science into internationally accepted standard methods with applications in CRA. As such, the model developed in this study demonstrates that qAOPs can become informative tools to determine the potential hazards arising from chemical exposure, and hence support actions to prevent or reduce the associated risks.

## 5 Conclusions

A Bayesian hierarchical multiparameter model was developed for a simplified AOP network for DNT derived from a topology analysis, combined with expert judgement. The modelling workflow achieved three goals: it dealt with missing values; accommodated unbalanced and correlated data; and followed the structure of a DAG to simulate the biological path. The model itself, derived from the workflow, can be used to predict the DNT potential of a compound as well as the probability of triggering each of the three upstream CKEs with the associated uncertainty resulting from different sources of data variability. In addition, the model can guide further data generation to better understand DNT mechanistically and support decision-making in the regulatory assessment of chemicals, e.g., by supporting the development of chemical categories and the application of read-across. The methodology can be applied to other endpoints of interest and can be updated to accommodate new evidence. Future directions include the addition of other biological paths and kinetic information to extend the applicability domain and utility as a qAOP model.

## Declaration of Competing Interest

The authors declare that they have no known competing financial interests or personal relationships that could have appeared to influence the work reported in this paper.
