# Supplementary material for: Probabilistic modelling of developmental neurotoxicity based on a simplified adverse outcome pathway network
Source: Comput Toxicol. 2022 Feb;21:100206. doi: 10.1016/j.comtox.2021.100206 (PMC8857173; doi:10.1016/j.comtox.2021.100206)
Supplement: Supplementary data 2 [file mmc2.docx]

**Supplementary Information**

**Probabilistic Modelling of a Simplified Adverse Outcome Pathway Network for Developmental Neurotoxicity**

Nicoleta Spînu^a^, Mark T.D. Cronin^a^, Junpeng Lao^b^, Anna Bal-Price^c^, Ivana Campia^c^, Steven J. Enoch^a^, Judith C. Madden^a^, Liadys Mora Lagares^d,e^, Marjana Novič^e^,
David Pamies^f,g^, Stefan Scholz^h^, Dan L. Villeneuve^i^, Andrew P. Worth^c^

^a^School of Pharmacy and Biomolecular Sciences, Liverpool John Moores University, Byrom Street, Liverpool,
L3 3AF, UK

^b^Department of Psychology, University of Fribourg, Fribourg CH-1700, Switzerland

^c^European Commission, Joint Research Centre (JRC), Ispra, Italy

^d^Jožef Stefan International Postgraduate School, 1000 Ljubljana, Slovenia

^e^Theory Department, Laboratory for Cheminformatics, National Institute of Chemistry, 1000 Ljubljana, Slovenia

^f^Department of Biomedical Science, University of Lausanne, Lausanne, Vaud, Switzerland

^g^Swiss Centre for Applied Human Toxicology (SCAHT), Switzerland

^h^Helmholtz-Centre for Environmental Research − UFZ, Department of Bioanalytical Ecotoxicology, Permoserstrasse 15, 04318 Leipzig, Germany

^i^National Health and Environmental Effects Research Laboratory, US Environmental Protection Agency, Duluth, MN 55804, Minnesota, USA

Corresponding Author: Andrew P. Worth; Tel: +39 0332 789566; E-mail: [andrew.worth@ec.europa.eu](mailto:andrew.worth@ec.europa.eu); European Commission, Joint Research Centre (JRC), Ispra, Italy

**Supplementary Figures**

[Figure S1. Diagram of the simplified AOP network on which the modelling is based. 3](#_Toc80776631)

[Figure S2. Overview of the distribution of the categorical variables, which were analysed by the Bayesian hierarchical model. 4](#_Toc80776632)

[Figure S3. Overview of the distribution of the continuous type of variables, which were analysed by the Bayesian hierarchical model. 5](#_Toc80776633)

[Figure S4. Visual representation of the correlation matrix between both categorical and continuous variables in the dataset. 6](#_Toc80776634)

[Figure S5. Visual representation of the distribution of the non-missing values (%) for all variables and the number of compounds of the dataset with non-missing information. 6](#_Toc80776635)

[Figure S6. Trace plots of the parameter estimates after the imputation of missing values of the Bayesian hierarchical model. 7](#_Toc80776636)

[Figure S7. Trace plots of the parameter estimates of the missing values of the Bayesian hierarchical model. 8](#_Toc80776637)

[Figure S8. Forest plot of the hyperpriors and priors of the inference sampling. 9](#_Toc80776638)

[Figure S9. Overview of the $\boldsymbol{\theta}\mathbf{s}$ likelihood linear predictions of each common key event. 10](#_Toc80776639)

[Figure S10. The predicted probabilities of ordered compounds for their potential to induce BDNF reduction. 11](#_Toc80776640)

[Figure S11. The predicted probabilities of ordered compounds for their potential to induce a decrease of synaptogenesis. 12](#_Toc80776641)

[Figure S12. The predicted probabilities of ordered compounds for their potential to inducing a decrease of neural network formation. 13](#_Toc80776642)


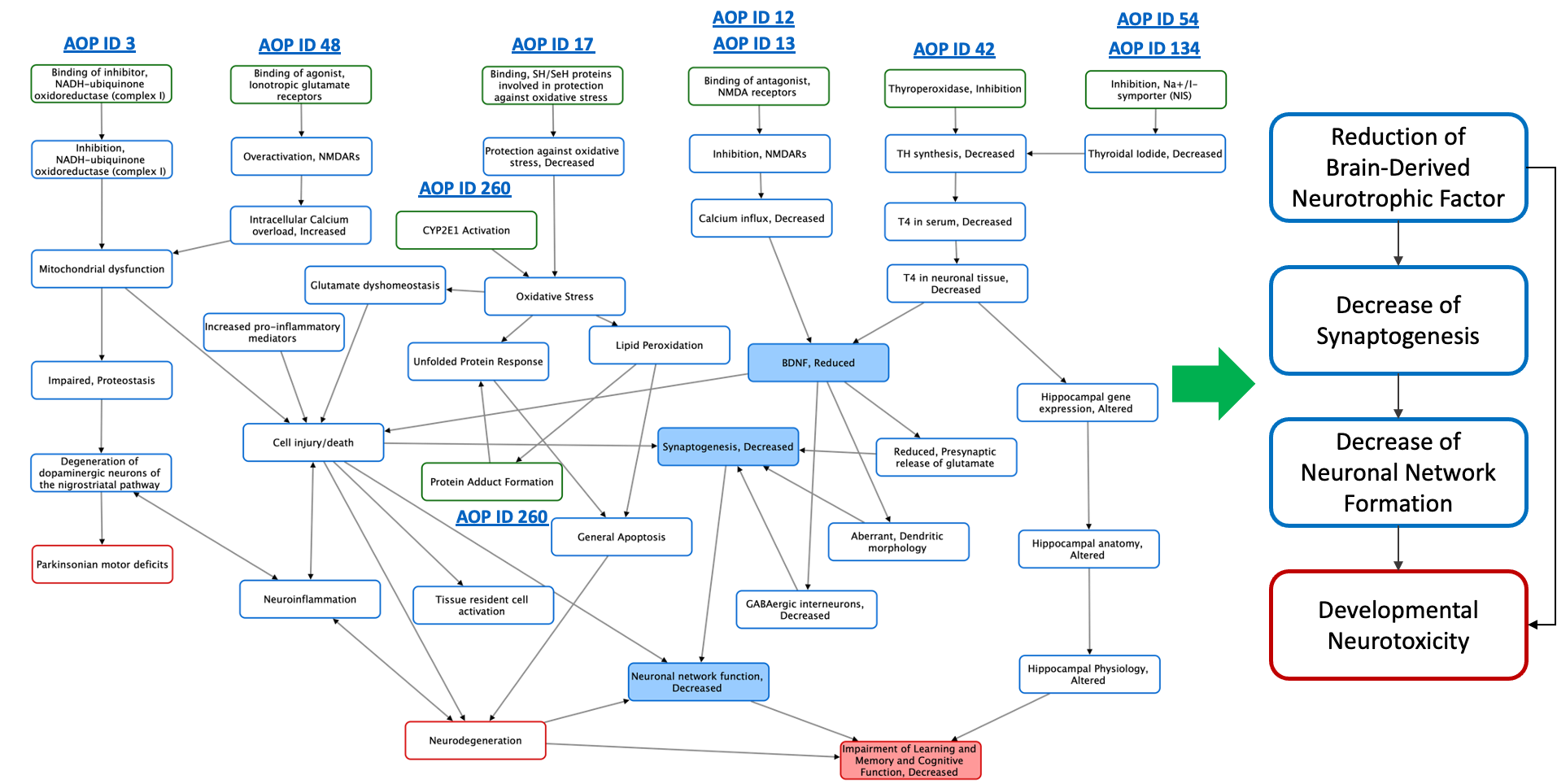


Figure S1. **Diagram of the simplified AOP network on which the modelling is based.**

The left side shows the derived network of nine AOPs for neurotoxicity containing adjacent key event relationships (KERs) taken from [1]. Green squares indicate a molecular initiating event (MIE), red squares indicate an adverse outcome (AO), and the red squares filled with red colour indicates the most common/highly connected AO. Blue squares indicate the most common/highly connected key events (KEs) chosen for the development of the predictive model (see text for further explanation regarding excluding Cell injury/death as a common/highly connected KE). Solid arrows indicate relationships between KEs that are adjacent. On the right side, the simplified biological path used for probabilistic modelling is presented.


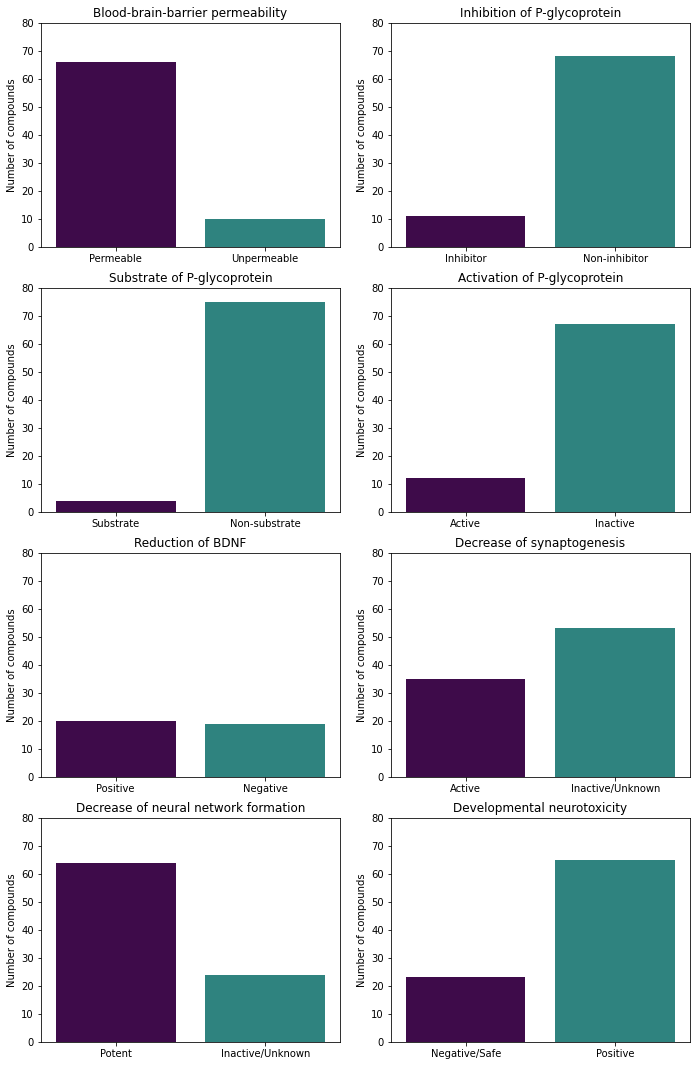


Figure S2. **Overview of the distribution of the categorical variables, which were analysed by the Bayesian hierarchical model.**

The figure shows the presence of unbalanced categories with the exception of the common key event (CKE) “decrease of synaptogenesis”. Missing information is not shown herein. The data sources are described in Table 1 and made available in Tables S2 and S3.


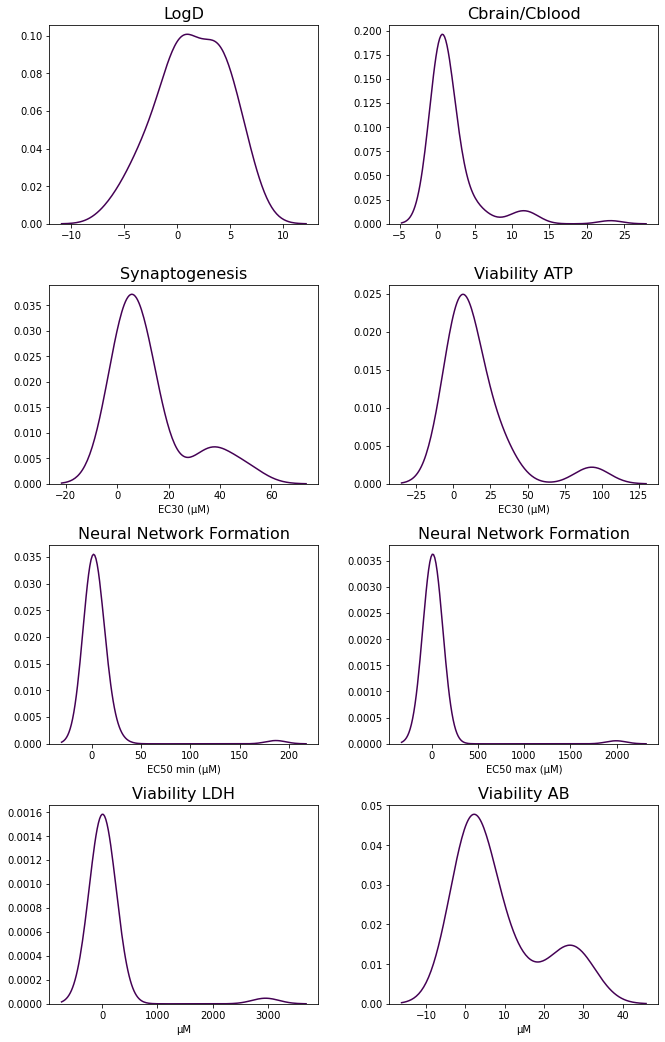


Figure S3. **Overview of the distribution of the continuous type of variables, which were analysed by the Bayesian hierarchical model.**

The data sources are described in Table 1 and made available in Tables S2 and S3. The EC_30_ and EC_50_ values were extracted from the publications referenced therein.


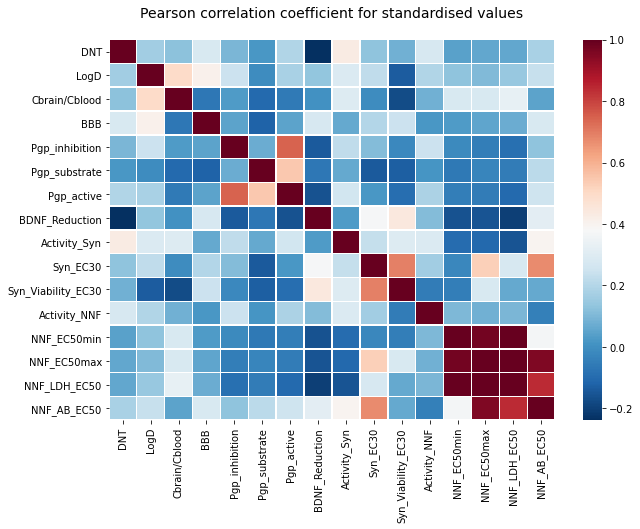


Figure S4. **Visual representation of the correlation matrix between both categorical and continuous variables in the dataset.**

This is based on the Pearson correlation coefficient for both predictors and outcomes. A value of one shows a total positive linear correlation.


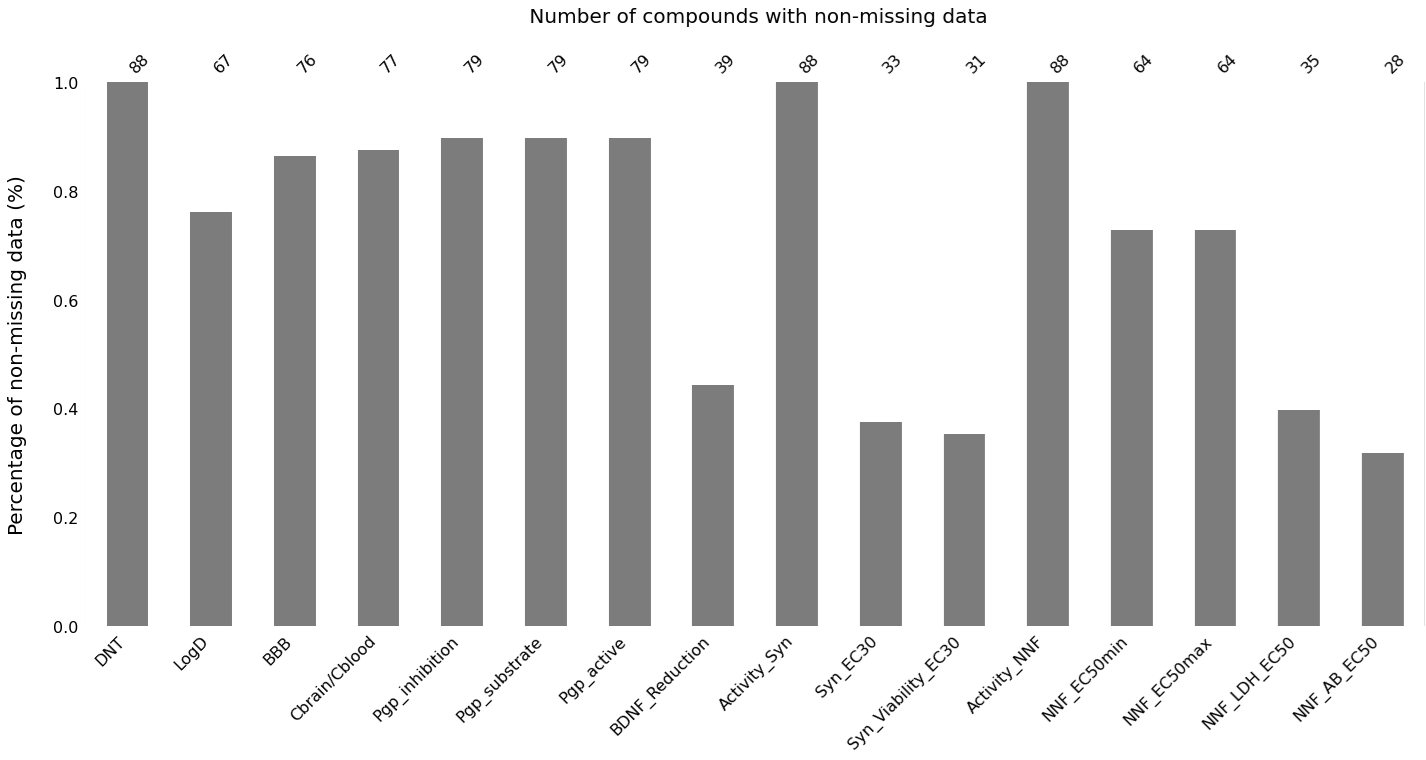


Figure S5. **Visual representation of the distribution of the non-missing values (%) for all variables and the number of compounds of the dataset with non-missing information**.

The missingness was present at all levels: in silico predictions since the dataset contained organic and inorganic compounds, and in vitro predictions, as not all compounds showed activity in both in vitro systems. It was calculated using the Missingno v.0.4.2 Python package [2].


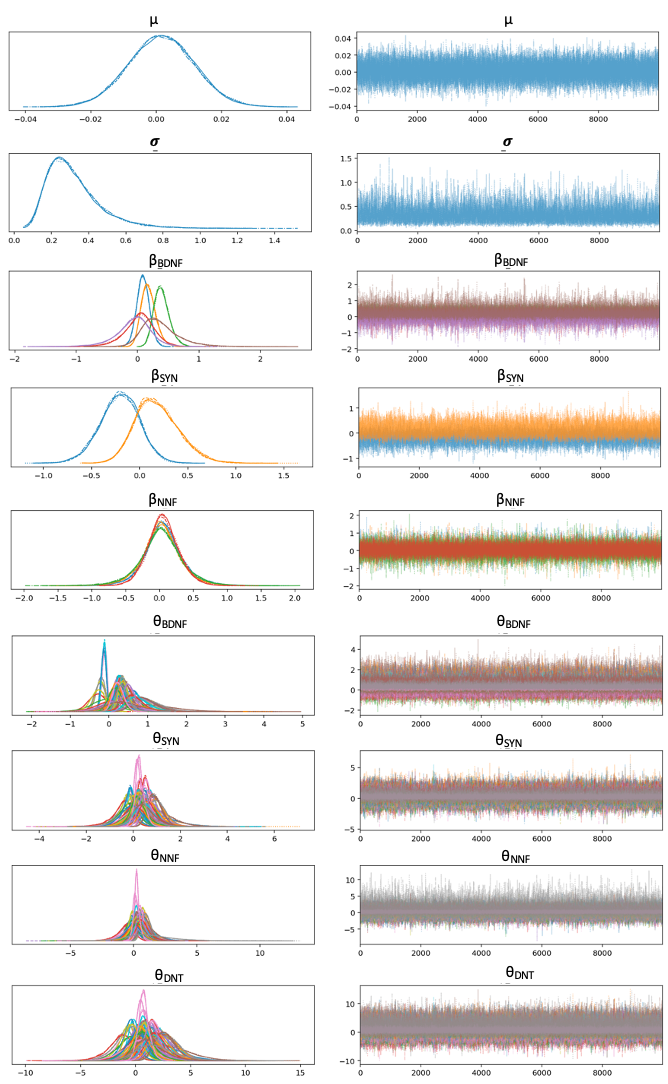


Figure S6. **Trace plots of the parameter estimates after the imputation of missing values of the Bayesian hierarchical model.**

Distributions of the sampled values for hyperpriors, i.e., common mean $\mu$ and standard deviation $\sigma$, and for priors, which are the coefficients describing each CKE ${{\beta_{BDNF},\beta}_{SYN},\beta}_{NNF}$, and multivariate regressions used to model predictions for each CKE $\theta_{BDNF},\theta_{SYN}, \theta_{NNF}$(Figure 2 shows the structure of the model that the sampling/inference step followed).

The left plot shows the estimated probability distribution for each independent trace. The right plot shows the actual sampling followed through the distribution. The colours indicate the set of the predictors on which the parameter was computed. For example, $\beta_{BDNF}$ described physicochemical properties including SLogP, blood-brain-barrier (two data sources) and activity against P-glycoprotein (three-classes, one-hot encoded), and hence, the six distributions coloured differently are presented (Figure 1 shows the type of information used for modelling purposes).


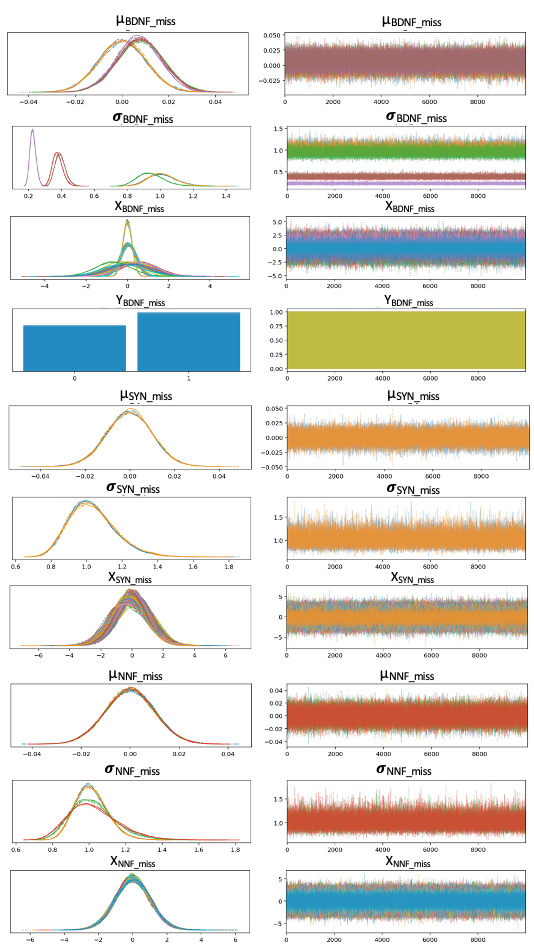


Figure S7. **Trace plots of the parameter estimates of the missing values of the Bayesian hierarchical model.**

The $X$ predictors that contained missing data were sampled from the specified prior distribution, mean $\mu$ and standard deviation $\sigma$ taking into account the data that described each CKE. The missing $Y$ outcomes were imputed from the posterior predictive distributions defined as a Bernoulli distribution.

The left plot shows the estimated probability distribution for each independent trace out of four traces run in total. The right plot shows the actual sampling followed through the distribution. The colours indicate the set of the predictors on which the parameter was computed.


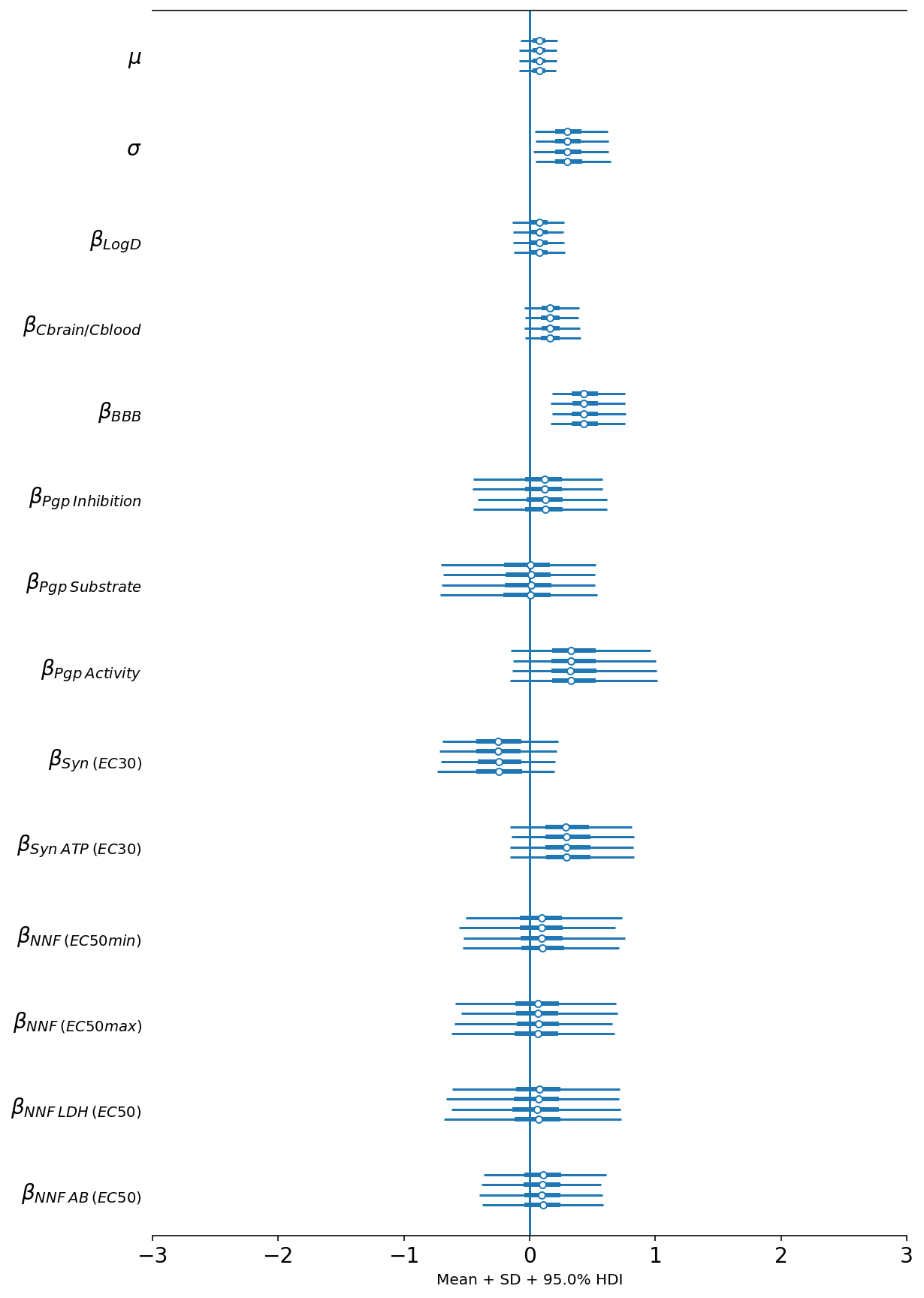


Figure S8. **Forest plot of the hyperpriors and priors of the inference sampling.**

It shows the results for each of the parameters computed on the data. The $\beta$ parameters are ordered following the order of the input data used to describe the corresponding CKE. The dot represents the mean, the thicker line is the standard deviation (SD), and the thin line represents the 95% of the Bayesian credible interval (CI), also known as the highest density interval (HDI). Each blue line corresponds to each independent chain (i.e., trace) out of four chains run in total.


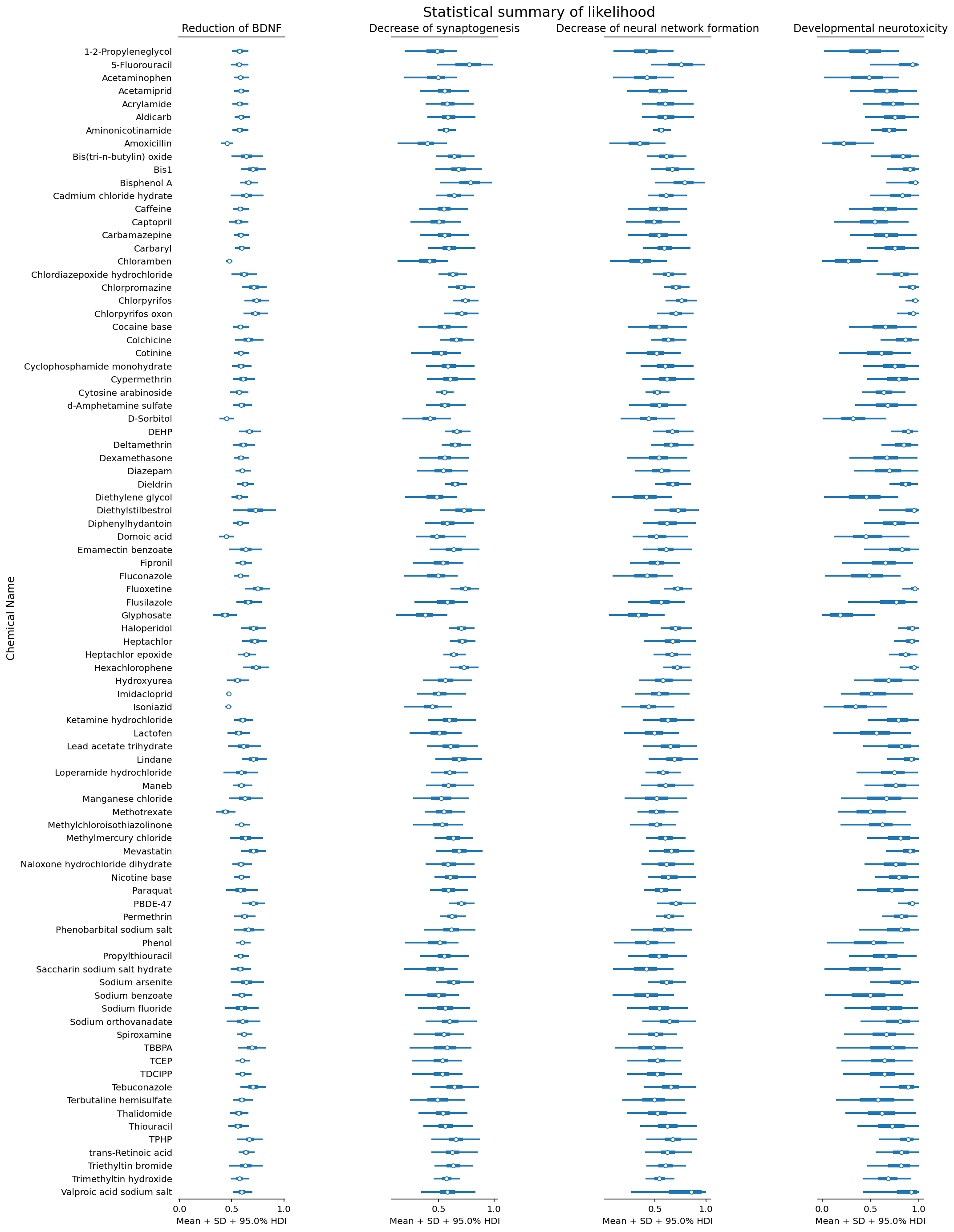


Figure S9. **Overview of the** $\boldsymbol{\theta}\mathbf{s}$ **likelihood linear predictions of each common key event.**

The dot represents the mean, the thicker line is the standard deviation (SD), and the thin line represents the 95% of the Bayesian credible interval (CI), also known as the highest density interval (HDI). A low mean predicted probability close to zero indicates that a chemical has a lower probability of inducing the corresponding CKE, whereas a high mean predicted probability closer to one indicates a higher probability of inducing the corresponding CKE. A larger HDI represents a higher level of uncertainty given by e.g., missingness. Compounds are ordered alphabetically.

*
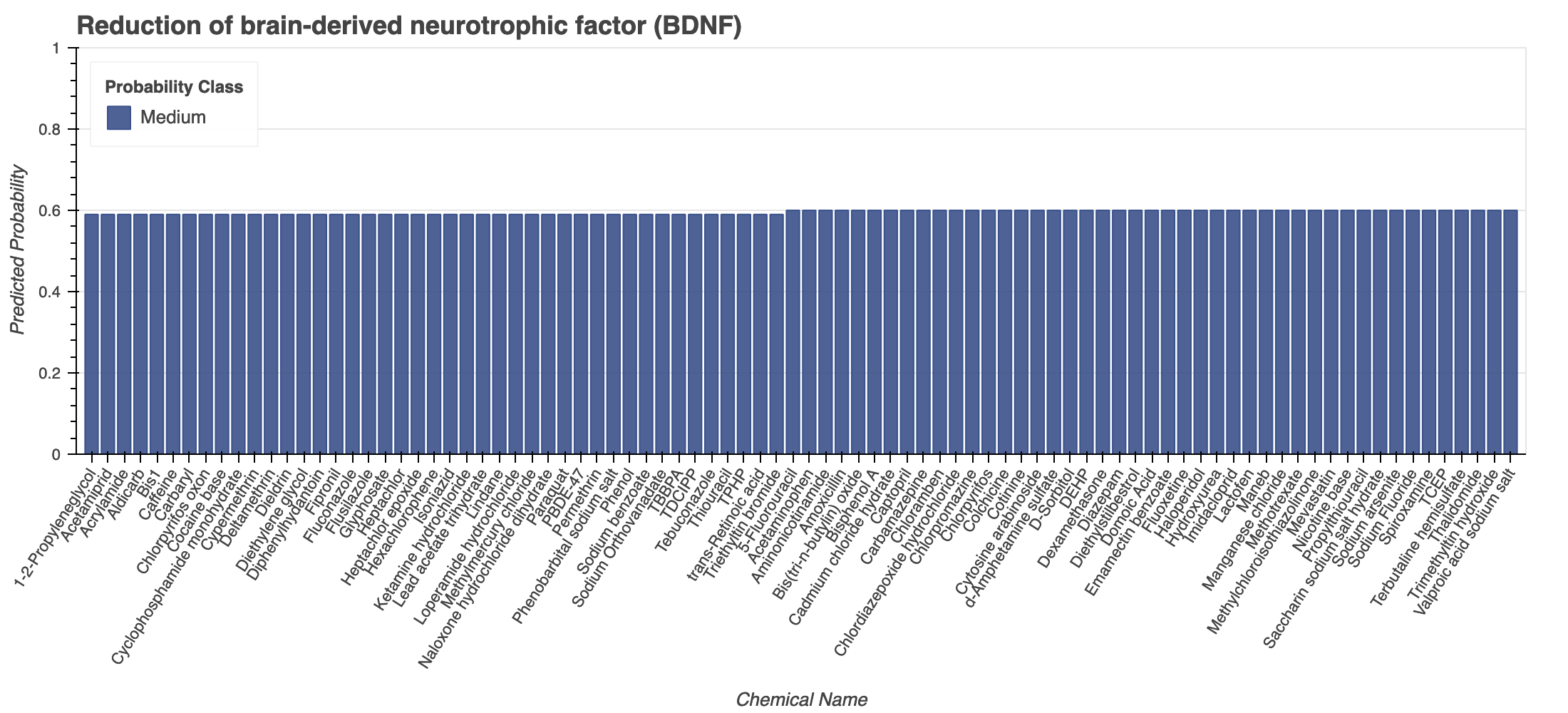
*

Figure S10. **The predicted probabilities of ordered compounds for their potential to induce BDNF reduction.**

The predicted probabilities are colour-coded based on two thresholds estimated from the results set to group the compounds for their high, medium and low probability. Herein, all the compounds have been predicted with a medium level of probability. An explanation can be because of the missing information for more than half of the compounds computed from the posterior distribution of the binary classification. Compounds were listed in order of increasing probability.

*
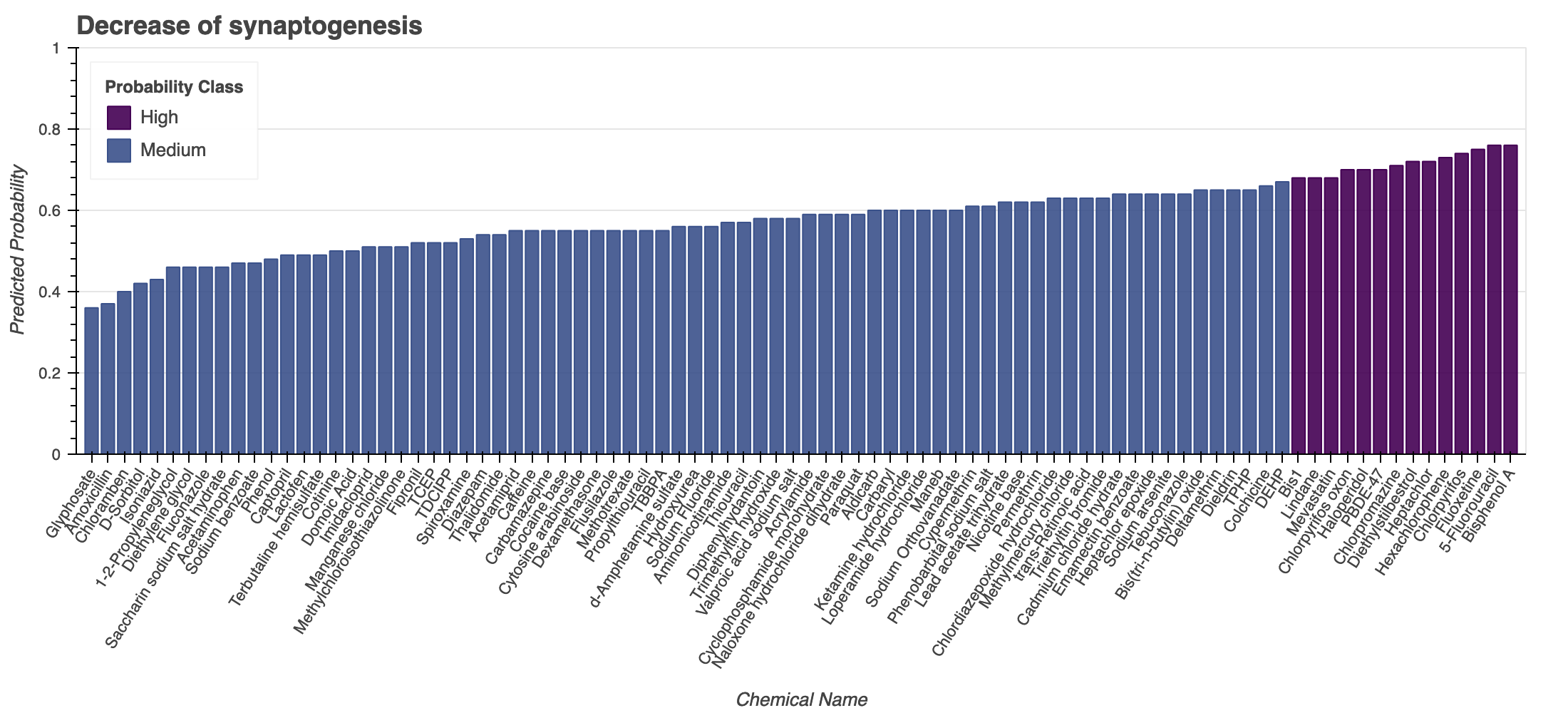
*

Figure S11. **The predicted probabilities of ordered compounds for their potential to induce a decrease of synaptogenesis.**

The predicted probabilities are colour-coded based on two thresholds estimated from the results set to group the compounds for their high, medium and low probability. Herein, the compounds have been predicted with a medium and high level of probability. Compounds were listed in order of increasing probability.


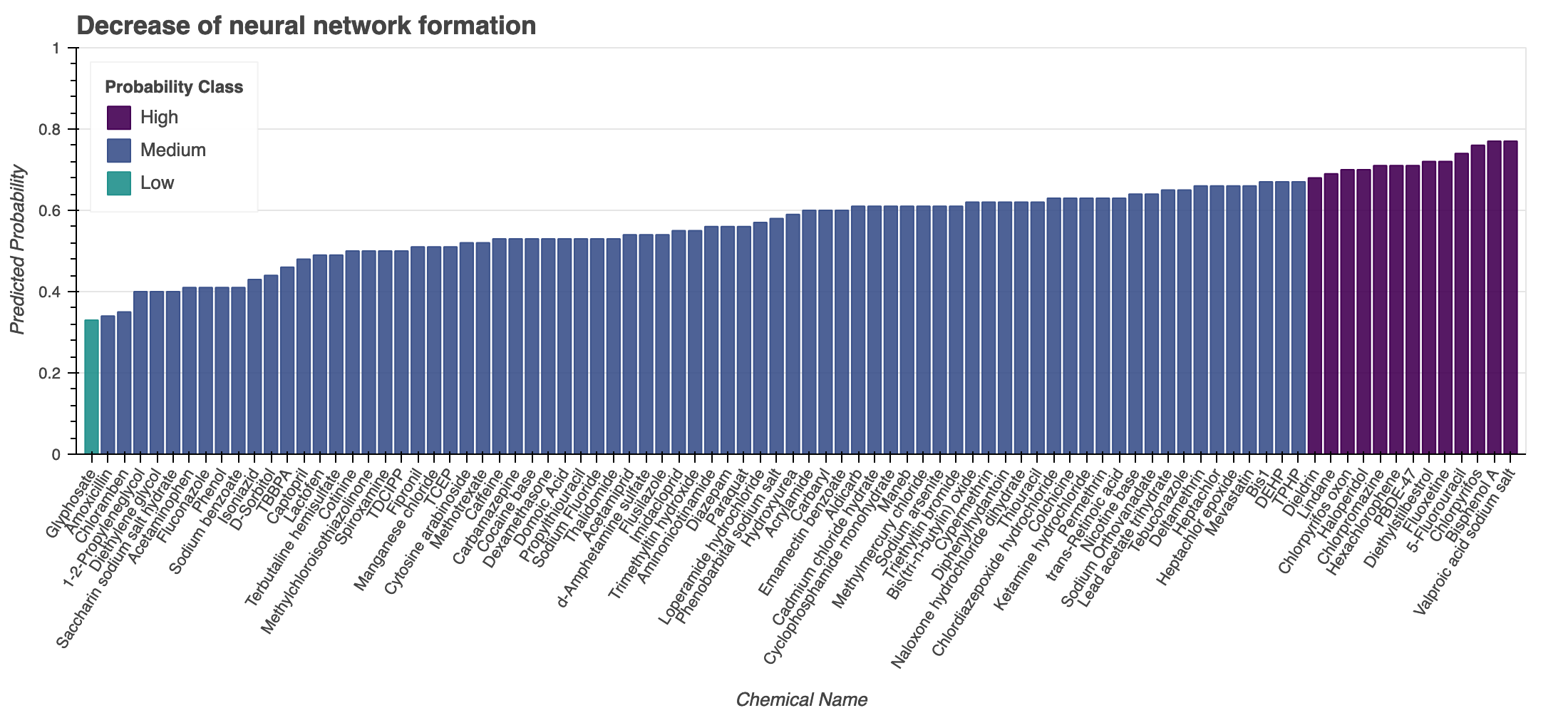


Figure S12. **The predicted probabilities of ordered compounds for their potential to inducing a decrease of neural network formation.**

The predicted probabilities are colour-coded based on two thresholds estimated from the results set to group the compounds for their high, medium and low probability. Compounds were listed in order of increasing probability.

**References**

1. Spînu, N., et al., *Development and analysis of an adverse outcome pathway network for human neurotoxicity.* Arch. Toxicol, 2019. **93**(10): p. 2759-2772.

2. Bilogur, A., *Missingno: a missing data visualization suite.* Journal of Open Source Software, 2018. **3(22)**(547).
