# Supplementary material for: Probabilistic modelling of developmental neurotoxicity based on a simplified adverse outcome pathway network
Source: Comput Toxicol. 2022 Feb;21:100206. doi: 10.1016/j.comtox.2021.100206 (PMC8857173; doi:10.1016/j.comtox.2021.100206)
Supplement: Supplementary data 4 [file mmc4.zip › PyMC3 Model.html]

PyMC3 Model


In [1]:

```
# --- Import libraries --- #
import pandas as pd
import numpy as np
from patsy import dmatrices, dmatrix
from scipy import stats
from scipy.special import expit as logistic
from sklearn.preprocessing import StandardScaler
from sklearn.impute import SimpleImputer

import theano
import theano.tensor as tt
import pymc3 as pm
import arviz as az
import arviz.labels as azl

import warnings
warnings.simplefilter(action="ignore")
%config InlineBackend.figure_format = 'retina'

print("Packages uploaded successfully!")
```

```
Packages uploaded successfully!
```

In [2]:

```
# --- Read data --- #
data = pd.read_csv('../data/machine_readable.csv')

# --- Standardise continuous values --- #
data_prep = data[['LogD', 'Cbrain/Cblood','Syn_EC30', 
                  'Syn_Viability_EC30', 'NNF_EC50min', 'NNF_EC50max', 'NNF_LDH_EC50', 'NNF_AB_EC50']]
sc = StandardScaler()
data_scaled = sc.fit_transform(data_prep)
data_scaled = pd.DataFrame(data_scaled)
data_scaled.columns = ['LogD', 'Cbrain/Cblood','Syn_EC30', 
                       'Syn_Viability_EC30', 'NNF_EC50min', 'NNF_EC50max', 'NNF_LDH_EC50', 'NNF_AB_EC50']

# Add other discrete values and details
discrete = data [['Chemical', 'CASRN', 'DNT', 'BBB', 'Pgp_inhibition', 'Pgp_substrate', 'Pgp_active', 
  'BDNF_Reduction', 'Activity_Syn', 'Activity_NNF']]
data = discrete.join(data_scaled, lsuffix="_left", rsuffix="_right")
```

In [3]:

```
# --- Define predictors and outcomes --- #

# BDNF given by continuous and discrete variables
x_bdnf = pd.DataFrame(data[['LogD', 'Cbrain/Cblood', 'BBB', 
                            'Pgp_inhibition', 'Pgp_substrate', 'Pgp_active']]).values
y_bdnf = pd.DataFrame(data[['BDNF_Reduction']]).values

# SYN
x_syn = pd.DataFrame(data[['Syn_EC30', 'Syn_Viability_EC30']]).values
Y_syn = pd.DataFrame(data[['Activity_Syn']]).values #Y because no missing values

# NNF
x_nnf = pd.DataFrame(data[['NNF_EC50min', 'NNF_EC50max', 'NNF_LDH_EC50', 'NNF_AB_EC50']]).values
Y_nnf = pd.DataFrame(data[['Activity_NNF']]).values #Y because no missing values

# x_dnt is given by causal relations BDNF->SYN->NNF->DNT<-BNDF
Y_dnt = pd.DataFrame(data[['DNT']]).values #Y because no missing values
```

In [4]:

```
# --- Mask missing data --- #
# BDNF
x_bdnf_missing = np.isnan(x_bdnf)
X_bdnf_train = np.ma.masked_array(x_bdnf, mask=x_bdnf_missing)
y_bdnf_missing = np.isnan(y_bdnf)
Y_bdnf_train = np.ma.masked_array(y_bdnf, mask=y_bdnf_missing)

# SYN
x_syn_missing = np.isnan(x_syn)
X_syn_train = np.ma.masked_array(x_syn, mask=x_syn_missing)

# NNF
x_nnf_missing = np.isnan(x_nnf)
X_nnf_train = np.ma.masked_array(x_nnf, mask=x_nnf_missing)
```

In [5]:

```
# --- Define and fit the model --- #
with pm.Model() as model_hierar:
    # Define hyperpriors
    mu_beta = pm.Normal('mu_beta', mu=0, sd=0.01) 
    sd_beta = pm.HalfNormal('sd_beta', sd=1)
    # Define priors
    beta_bdnf = pm.Normal('beta_bdnf', mu=mu_beta, sd=sd_beta, shape=(6,1))
    beta_syn = pm.Normal('beta_syn', mu=mu_beta, sd=sd_beta, shape=(2,1))
    beta_nnf = pm.Normal('beta_nnf', mu=mu_beta, sd=sd_beta, shape=(4,1))
    
    
    # Imputation of X missing values for BDNF
    Xmu_bdnf = pm.Normal('Xmu_bdnf', mu=0, sd=0.01, shape=(1,6))
    Xsigma_bdnf = pm.HalfNormal('Xsigma_bdnf', sd=1, shape=(1,6))
    X_bdnf_modelled = pm.Normal('X_bdnf_modelled', 
                                         mu=Xmu_bdnf, sigma=Xsigma_bdnf, observed=X_bdnf_train)

    # Likelihood for BDNF
    # SLogP, Cbrain/Cblood, BBB, Pgp->BDNF
    lp_bdnf = pm.Deterministic('lp_bdnf', pm.math.dot(X_bdnf_modelled, beta_bdnf))
    y_obs_bdnf = pm.Bernoulli('y_obs_bdnf', logit_p=lp_bdnf, observed=Y_bdnf_train)
    
    # Imputation of X missing values for SYN
    Xmu_syn = pm.Normal('Xmu_syn', mu=0, sd=0.01, shape=(1,2))
    Xsigma_syn = pm.HalfNormal('Xsigma_syn', sd=1, shape=(1,2))
    X_syn_modelled = pm.Normal('X_syn_modelled',
                                 mu=Xmu_syn, sigma=Xsigma_syn, observed=X_syn_train)

    # Likelihood for SYN
    # BDNF->SYN
    lp_syn = pm.Deterministic('lp_syn', lp_bdnf + pm.math.dot(X_syn_modelled, beta_syn))
    y_obs_syn = pm.Bernoulli("y_obs_syn", logit_p=lp_syn, observed=Y_syn)
    
    # Imputation of X missing values for NNF
    Xmu_nnf = pm.Normal('Xmu_nnf', mu=0, sd=0.01, shape=(1,4))
    Xsigma_nnf = pm.HalfNormal('Xsigma_nnf', sd=1, shape=(1,4)) 
    X_nnf_modelled = pm.Normal('X_nnf_modelled',
                                 mu=Xmu_nnf, sd=Xsigma_nnf, observed=X_nnf_train)
   
    # Likelihood for NNF
    # BDNF->SYN->NNF
    lp_nnf = pm.Deterministic('lp_nnf', lp_syn + pm.math.dot(X_nnf_modelled, beta_nnf))
    y_obs_nnf = pm.Bernoulli("y_obs_nnf", logit_p=lp_nnf, observed=Y_nnf)
    
    # Define causal relationships for DNT
    lp_dnt = pm.Deterministic('lp_dnt', lp_bdnf + lp_syn + lp_nnf)
    y_obs_dnt = pm.Bernoulli('y_obs_dnt', logit_p=lp_dnt, observed=Y_dnt)

# Checking the proposed structure of model
model_hierar.check_test_point()
```

Out[5]:

```
mu_beta                      3.69
sd_beta_log__               -0.77
beta_bdnf                   -4.16
beta_syn                    -1.39
beta_nnf                    -2.77
Xmu_bdnf                    22.12
Xsigma_bdnf_log__           -4.62
X_bdnf_modelled_missing      0.00
y_obs_bdnf_missing           0.00
Xmu_syn                      7.37
Xsigma_syn_log__            -1.54
X_syn_modelled_missing       0.00
Xmu_nnf                     14.74
Xsigma_nnf_log__            -3.08
X_nnf_modelled_missing       0.00
X_bdnf_modelled           -552.12
y_obs_bdnf                 -61.00
X_syn_modelled            -172.26
y_obs_syn                  -61.00
X_nnf_modelled            -394.00
y_obs_nnf                  -61.00
y_obs_dnt                  -61.00
Name: Log-probability of test_point, dtype: float64
```

In [6]:

```
# --- Run inferences and compute posterior distributions --- #
with model_hierar:
    trace_hierar = pm.sample(cores=4, draws=10000, nuts ={'target_accept':0.90})
    #Predictions
    posterior_hierar = pm.sample_posterior_predictive(trace_hierar)
```

```
Multiprocess sampling (4 chains in 4 jobs)
CompoundStep
>NUTS: [X_nnf_modelled_missing, Xsigma_nnf, Xmu_nnf, X_syn_modelled_missing, Xsigma_syn, Xmu_syn, X_bdnf_modelled_missing, Xsigma_bdnf, Xmu_bdnf, beta_nnf, beta_syn, beta_bdnf, sd_beta, mu_beta]
>BinaryGibbsMetropolis: [y_obs_bdnf_missing]
```

100.00% [44000/44000 02:53<00:00 Sampling 4 chains, 0 divergences]

```
Sampling 4 chains for 1_000 tune and 10_000 draw iterations (4_000 + 40_000 draws total) took 186 seconds.
The number of effective samples is smaller than 10% for some parameters.
```

100.00% [40000/40000 02:56<00:00]

In [7]:

```
%load_ext watermark
%watermark -n -u -v -iv -w
```

```
arviz  0.11.0
theano 1.0.4
pymc3  3.9.3
numpy  1.19.1
pandas 1.1.1
last updated: Fri Aug 06 2021 

CPython 3.8.5
IPython 7.18.1
watermark 2.0.2
```
